# Meta-analysis of robotic versus open pancreaticoduodenectomy in all patients and pancreatic cancer patients

**DOI:** 10.3389/fsurg.2022.989065

**Published:** 2022-10-11

**Authors:** Yibo Fu, Jiangdong Qiu, Yiqi Yu, Danning Wu, Taiping Zhang

**Affiliations:** ^1^ Department of General Surgery, Peking Union Medical College Hospital, Chinese Academy of Medical Sciences and Peking Union Medical College, Beijing, China; ^2^ Clinical Immunology Center, Chinese Academy of Medical Sciences and Peking Union Medical College, Beijing, China

**Keywords:** robotic pancreaticoduodenectomy, open pancreaticoduodenectomy, pancreatic cancer, outcome, meta-analysis

## Abstract

**Purposes:**

To compare perioperative outcomes of robotic pancreaticoduodenectomy (RPD) to open pancreaticoduodenectomy (OPD) using evidence from cohort studies.

**Methods:**

Outcomes of interest include operative time, blood loss, R0 resection rate, lymph nodes harvested, overall complication rate, pancreatic fistula rate, delayed gastric emptying rate and 90-day mortality.

**Results:**

6 prospective studies and 15 retrospective studies were included. Five of these studies were limited to patients with pancreatic cancer. Operative time was significantly longer in RPD (WMD: 64.60 min; 95% CI: 26.89 to 102.21; *p* = 0.001). Estimated blood loss was lower in RPD (WMD: −185.44 ml; 95% CI: −239.66 to −131.21; *p* < 0.001). Overall complication rates (OR: 0.66; 95% CI: 0.44 to 0.97; *p* < 0.001) and pancreatic fistula rate (OR: 0.67; 95% CI: 0.55 to 0.82; *p* < 0.001) were both lower in RPD. Length of hospital stay was longer in OPD (WMD: −1.90; 95% CI: −2.47 to −1.33). 90-day mortality was lower in RPD [odds ratio (OR): 0.77; 95% CI: 0.45 to 0.95; *p* = 0.025].

**Conclusion:**

At current level of evidence, RPD is a safer alternative than OPD with regard to post-operative outcomes and blood loss. However, in terms of oncological outcomes RPD show no advantage over OPD, and the cost of RPD was higher. In general, RPD is now considered a reliable technology, but high-quality randomized controlled trial (RCT) studies are still needed to support this conclusion.

## 1. Introduction

Pancreaticoduodenectomy (PD) has been universally accepted to be indicated in benign or malignant lesions of the pancreatic head, duodenum, and distal common bile duct. In 1994, Gagner reported the first laparoscopic pancreaticoduodenectomy, since when minimally invasive pancreaticoduodenectomy (MIPD) are increasingly being performed over the world (1). The development of the Da Vinci robotic platform takes MIPD a step further. Laparoscopic surgery has some shortcomings compared to robotic surgery, including limited vision and flexibility. And this contributed to the popularity of robotic surgery over the world (2). The first case of robotic-assistant pancreaticoduodenectomy (RAPD) was reported in 2007, and since then many studies have compared the safety and efficacy between open pancreaticoduodenectomy (OPD) and robotic pancreaticoduodenectomy (RPD). There have been several meta-analyses evaluating the effect between OPD and RPD. However, robotic surgery technology developed rapidly in these years, and the studies used in the existing meta-analyses are not new enough. Therefore, we focused on those studies published in the last 5 years (in or after 2016) to provide high-quality evidence for further clinical practice.

## 2. Methods

### 2.1. Literature-search strategy

A systematic review of the literature was performed in PubMed and Web of Science from January 2016 to October 2021. These key words were used: robot, robotic, robotic-assisted, open, and pancreaticoduodenectomy. Studies included should fulfill the following PICOS criteria in our meta-analyses. P (patients): Male or female patients with a benign or malignant disease that requires elective PD; I (intervention): RPD; C (control): OPD; O (outcome): At least 1 of the interested outcomes; S (study design): randomized controlled trials (RCTs) and observative studies.

References of the acquired articles were manually searched to broaden the search. When multiple researches describing the same population were published, the most complete or recent research was used.

### 2.2. Inclusion and exclusion criteria

The inclusion criteria were as followed: (1) comparative study of RPD and OPD; (2) papers written in English; (3) papers published in or after 2016. Abstracts, case reports, reviews, letters to the editor, non-comparative studies, and articles without available data were excluded.

### 2.3. Data extraction and outcome of interest

All references were reviewed and evaluated by two researchers independently. Only full-length articles were eligible for extraction. The following data of included articles were extracted: first author, year of publication, study design, number of operated subjects, operative time, blood loss, R0 resection rate, lymph nodes harvested, overall complication rate, pancreatic fistula rate, delayed gastric emptying and 90-day mortality.

### 2.4. Quality assessment

The Newcastle–Ottawa Scale (NOS) was used to evaluate the methodological quality of non-randomized studies. Scores of each observational study range from 0 to 9, and studies having six or more stars were considered to be high-quality studies.

### 2.5. Statistical analysis

This meta-analysis was performed using Stata MP 16.0 software. The odds ratios (OR) and the weighted mean difference (WMD) with a 95% confidence interval (95% CI) were used to estimate dichotomous and continuous variables, respectively. *p *< 0.05 was considered statistically significant. For studies that reported continuous data as median and range values (or quartile and median), the standard deviations were calculated using the method described by Luo et al. (3). Heterogeneity was evaluated by the Chi-square test, and *p* < 0.100 was considered significant. *I*^2^ values were used for the evaluation of statistical heterogeneity. An *I*^2^ value of 50% or more indicated the presence of heterogeneity. The fixed effect model (FEM) and random effect model (REM) were used based on the value of *I*^2^. FEM was used in the case of *I*^2^ < 50% while REM was adopted in the case of *I*^2^ > 50%.

## 3. Result

### 3.1. Literature-search results

**Figure 1:**
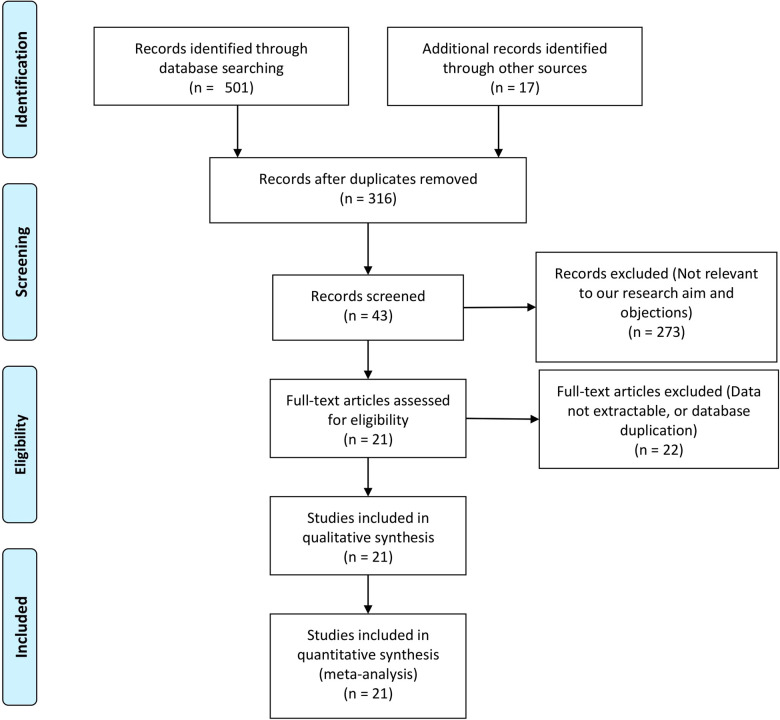
Flow diagram of the selection progress.

The first search strategy generated 518 studies. 21 articles including 5,756 patients (2,561 cases for RPD and 3,285 cases for OPD) fulfilled the predefined inclusion criteria and were included in this meta-analysis (4–24). All studies were non-RCTs, of which 6 studies were prospective while 15 studies were retrospective. The selection process is shown in Figure 1.

### 3.2. Study characteristics and quality assessment

The study characteristics and study quality are shown in Table 1. We screened articles published in 2016 and beyond. This is a worldwide meta-analysis, in which eight articles are from America, six articles are from China, four articles are from Italy, two articles are from Korea, and one article is from Spain. In most studies the robotic surgery group carried out RPD, while in four studies RAPD were used, where robots were only involved in some parts of the surgery. In five studies patients were limited to pancreatic cancer, while in other studies the indication for surgery were wide, including benign and malignant disease. All the studies were of high quality according to the Newcastle–Ottawa Scale (NOS) (Supplementary Table S1).

**Table 1:** Characteristics of the included studies and quality assessment.

Article	Country	Design	Number of patients (robotic)	Number of patients (open)	Quality score	Robotic technique	Indication for surgery (benign or malignant disease)
Emanuele 2018	Italy	Retrospective	24	26	6	RPD	M
Hassan 2021	America	Retrospective	310	310	8	RPD	M
Shyr 2021	China	Prospective	65	65	6	RPD	M
Maria 2020	America	Retrospective	38	38	7	RPD	M
Weng 2020	China	Retrospective	105	210	8	RAPD	M
Amer 2016	America	Retrospective	211	817	6	RPD	B/M
Matthew 2016	America	Retrospective	152	152	7	RPD	B/M
Mejia 2020	America	Retrospective	102	54	6	RPD	B/M
Wang 2018	China	Prospective	87	87	8	RPD	B/M
Kim 2018	Korea	Retrospective	51	186	7	RPD	B/M
Varley 2018	America	Retrospective	133	149	7	RPD	B/M
Cai 2019	America	Prospective	460	405	8	RPD	B/M
Paolini 2021	Italy	Retrospective	65	53	6	RPD	B/M
Benedetto 2018	Spain	Prospective	17	17	7	RPD	B/M
Marino 2019	Italy	Prospective	35	35	8	RAPD	B/M
Shi 2021	China	Retrospective	187	187	8	RAPD	B/M
Bencini 2020	Italy	Retrospective	35	35	8	RPD	B/M
Hyeyeon 2020	Korea	Retrospective	55	55	7	RAPD	B/M
Oosten 2020	America	Retrospective	96	192	8	RPD	B/M
Shyr 2020	China	Retrospective	284	169	6	RPD	B/M
Wang 2021	China	Prospective	49	43	7	RPD	B/M

Patients’ baseline characteristics are summarized in Table 2.

**Table 2:** Comparison of patients’ baseline characteristics in robotic vs. open pancreaticoduodenectomy.

Article	Age (years)		Gender (male) (%)		BMI		Tumor diameter (cm)		Preoperative CA 199	
	RPD	OPD	RPD	OPD	RPD	OPD	RPD	OPD	RPD	OPD
Emanuele 2018	65 (58.5–74.75)	72.5 (59.75–78.75)	50.0	54.1	23.1 ± 3.2	24.1 ± 3.1	2.7 ± 0.6	2.7 ± 0.9	353.3 ± 528.6	1362.7 ± 4497
Hassan 2021	66 ± 21.3	68.1 ± 19.3	50.1	51.1	NM	NM	NM	NM	NM	NM
Shyr 2021	66 ± 13	66 ± 11	52.3	40.0	24 ± 4	22 ± 3	3.1 ± 0.8	3.1 ± 0.7	NM	NM
Maria 2020	66 (38–84)^a^	68 (42–81)^a^	42.1	42.1	24.7 (19.6–39.1)^a^	25.7 (15.8–44.8)^a^	3 (0.5–6)^a^	2.9 (0.9–7)^a^	NM	NM
Weng 2020	63 (57–68)	64 (58–70)	61.7	65.9	22.8 ± 2.8	22.6 ± 3.1	3 (2.2–3.5)	3.0 (2.3–3.8)	144.4 (40.1–375.4)	153.4 (46.0–505.2)
Amer 2016	67 (15–86)^a^	65 (15–93)^a^	52.9	55.5	27.5 (18.1–47.6)^a^	26.1 (14.7–85.5)^a^	2.5 (0.1–26.0)^a^	2.9 (0–5.0)^a^	NM	NM
Matthew 2016	NM	NM	NM	NM	NM	NM	NM	NM	NM	NM
Mejia 2020	66 ± 10.6	61.7 ± 14.1	52	55.6	NM	NM	3.4 ± 1.6	3.7 ± 2.1	NM	NM
Wang 2018	NM	NM	50	56.7	NM	NM	NM	NM	NM	NM
Kim 2018	60.7 ± 11.9	65.4 ± 10.1	47.1	58.1	22.7 ± 2.5	24.0 ± 3.1	NM	NM	NM	NM
Varley 2018	66.3 ± 10.6	67.0 ± 10.5	48	53	27.5 ± 6.1	26.7 ± 5.6	NM	NM	NM	NM
Cai 2019	66.5 ± 11.0	67.5 ± 10.7	55	52.1	27.8 ± 5.8	27.2 ± 5.9	NM	NM	NM	NM
Paolini 2021	70 (42–85)^a^	73 (45–91)^a^	50.9	53.8	26 (17–33)^a^	23 (14–33)^a^	2.3 (0.7–6)^a^	2.5 (0.6–8.2)^a^	85.0 (1.6–1,617.0)^a^	132.3 (1.6–91,000.0)^a^
Benedetto 2018	66.8 ± 9.5	61.4 ± 11.9	47.1	58.8	23.8 ± 4.1	24.6 ± 3.36	24.1 ± 5.4	24.8 ± 6.1	NM	NM
Marino 2019	60.4 (43–72)^a^	62.3 (45–73)^a^	54.3	42.9	23.8 (19.4–30.9)^a^	23.5 (18.8–28.1)^a^	2.35 (1.6–3.4)^a^	2.22 (1.2–3.5)^a^	NM	NM
Shi 2021	60.9 ± 11.4	60.1 ± 10.8	58.3	57.2	NM	NM	2.7 ± 1.1	2.7 ± 1.3	NM	NM
Bencini 2020	70.5 (42–85)^a^	69 (50–88)^a^	56.3	45.7	26 (18–32)^a^	24 (18–38)^a^	30 (18–40)^a^	37 (2–51)^a^	143 (2–1,617)^a^	70 (2–2,617)^a^
Hyeyeon 2020	58.6 ± 8.3	59.9 ± 13.4	47.3	54.5	23.7 ± 2.8	23.9 ± 3.6	2.6 ± 1.2	2.6 ± 1.8	NM	NM
Oosten 2020	67 (60–73)	67 (58–73)	NM	NM	26 (23–30)	27 (23–29)	NM	NM	NM	NM
Shyr 2020	65 ± 12	64 ± 11	53.3	53.5	24 ± 4	23 ± 3	3.2 ± 1.5	3.7 ± 2.5	NM	NM
Wang 2021	64.7 ± 11.8	64.8 ± 11.6	51.9	53.4	27.7 ± 5.6	27.4 ± 5.8	NM	NM	NM	NM

BMI, body mass index; expressed in mean ± SD and median (IQR).

^a^ Expressed in median (range).

### 3.3. Meta-analysis results

All 21 studies were included in this meta-analysis. The summarized result of meta-analysis is shown in Table 3.

**Table 3:** Outcomes of the included studies.

Outcomes	Studies, *n*	RPD	OPD	WMD/OR (95% CI)	*p* value	Heterogeneity	
						*I* ^2^	Tau^2^
Intraoperative outcomes							
Operative time	17	1,924	2,690	64.60 (26.89 to 102.21)	0.001	0.978	
Estimated blood loss	14	1,604	1,583	−185.44 (−239.66 to −131.21)	<0.001	0.927	
Oncological outcomes							
Lymph nodes harvested	13	1,337	1,699	1.13 (−0.27 to 2.54)	0.115	0.828	4.69
R0 resection	10	955	1,026	1.02 (0.79 to 1.30)	0.889	0	*n*
Post-operative outcomes							
Overall complication rates	13	1,192	1,856	0.66 (0.44 to 0.97)	<0.001	0.762	0.3524
Pancreatic fistula	13	1,938	2,104	0.67 (0.55 to 0.82)	<0.001	0.269	*n*
Length of stay	20	2,496	3,220	−1.90 (−2.47 to −1.33)	<0.001	0.685	0.6432
90-day mortality	12	1,841	2,591	0.77 (0.45 to 0.95)	0.025	0.038	*n*

#### 3.3.1. Intraoperative outcomes

##### 3.3.1.1. Operative time

**Figure 2:**
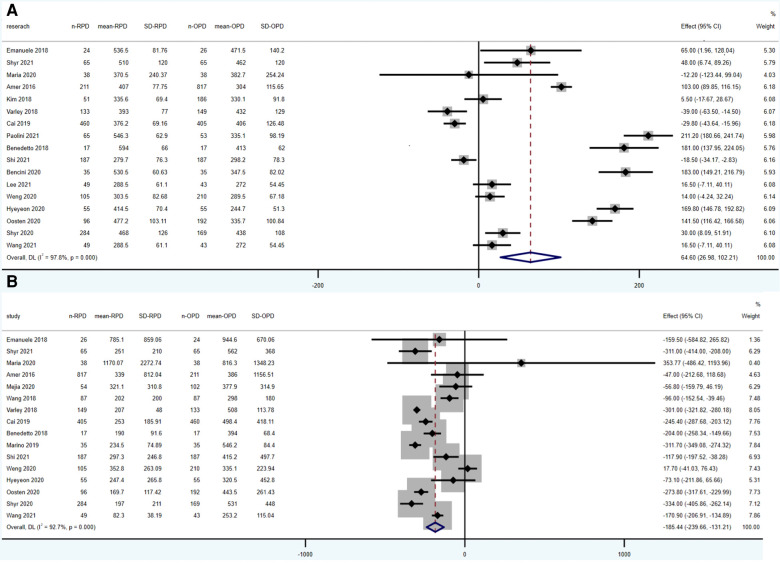
Meta-analysis of intraoperative outcomes: (**A**) operative time. (**B**) Estimated blood loss.

Operative time was reported in 17 studies (1,924 RPD vs. 2,690 OPD). According to the results of this meta-analysis, operative time was significantly longer in RPD group (WMD: 64.60 min; 95% CI: 26.89 to 102.21; *p* = 0.001), with high heterogeneity (*I*^2^ = 97.8%; Tau^2^ = 2133.45) in the REM (Figure 2A).

##### 3.3.1.2. Estimated blood loss

Estimated blood loss was reported in 14 studies (1,604 RPD vs. 1,583 OPD) and was significantly lower in RPD (WMD: −185.44 ml; 95% CI: −239.66 to −131.21; *p* < 0.001) with high among-study statistical heterogeneity (*I*^2^ = 92.7%; Tau^2^ = 3152.37) (Figure 2B).

#### 3.3.2. Oncological outcomes

##### 3.3.2.1. Lymph nodes harvested

**Figure 3:**
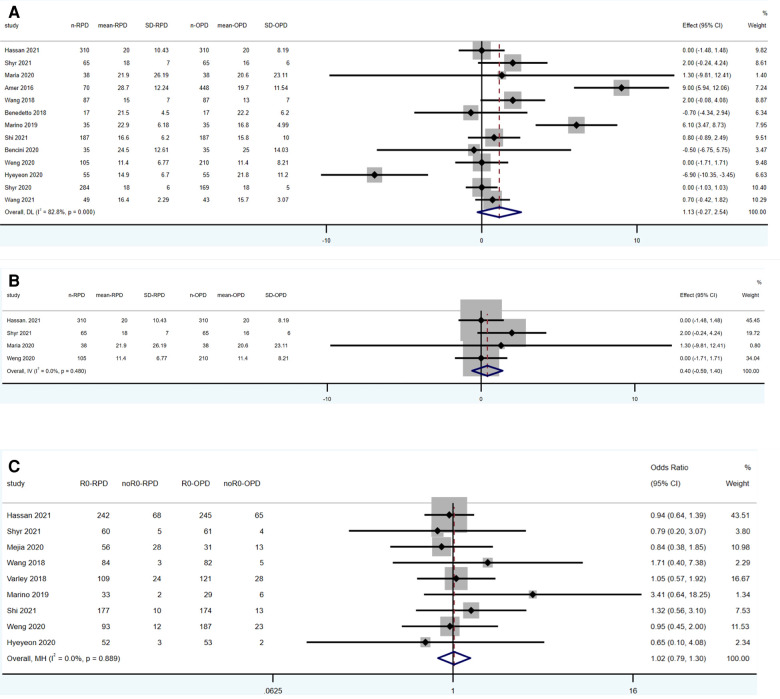
Meta-analysis of oncological outcomes: (**A**) lymph nodes harvested. (**B**) Lymph nodes harvested (in pancreatic cancer). (**C**) R0 resection.

13 studies reported the results of lymph nodes harvested (1,337 RPD vs. 1,699 OPD). No statistically significant differences were found between the two groups (WMD: 1.13; 95% CI: −0.27 to 2.54; *p *= 0.115), with a high heterogeneity (*I*^2^ = 82.8%, Tau^2^ = 4.69) in the REM (Figure 3A).

##### 3.3.2.2. Lymph nodes harvested (in pancreatic cancer)

Four studies reporting the results of lymph nodes harvested are limited in pancreatic cancer patients (518 RPD vs. 623 OPD). No statistically significant differences were found between the two groups (WMD: 0.4; 95% CI: −0.59 to 1.40; *p *= 0.425), with a low heterogeneity (*I*^2^ = 0%) in the FEM (Figure 3B).

##### 3.3.2.3. R0 resection

Ten studies reported the results of lymph nodes harvested (955 RPD vs. 1,026 OPD). No statistically significant differences were found between the two groups (OR: 1.02; 95% CI: 0.79 to 1.30; *p* = 0.889), with a low heterogeneity (*I*^2^ = 0%) in the FEM (Figure 3C).

#### 3.3.3. Post-operative outcomes

##### 3.3.3.1. Overall complication rates

**Figure 4:**
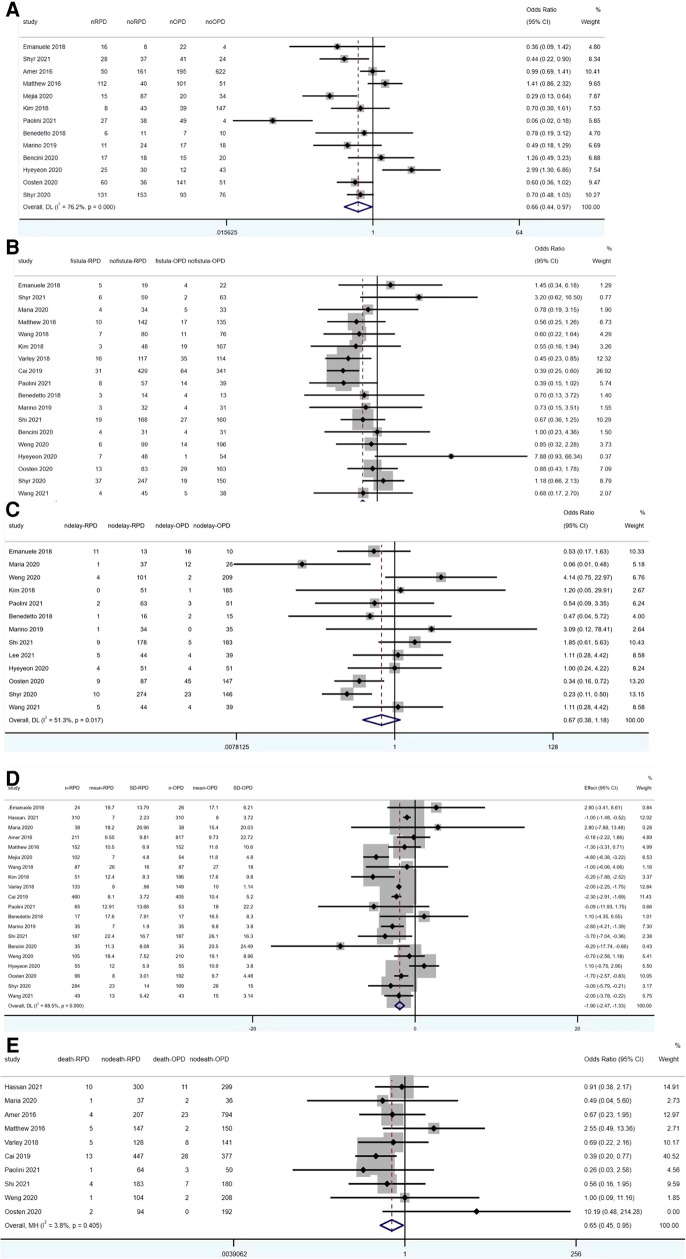
Meta-analysis of post-operative outcomes: (**A**) overall complication rates. (**B**) Pancreatic fistula. (**C**) Delayed gastric emptying. (**D**) Length of stay. (**E**) 90-day mortality.

Overall complication rate was reported in 13 studies (1,192 RPD vs. 1,856 OPD) and was significantly lower in RPD (OR: 0.66; 95% CI: 0.44 to 0.97; *p* < 0.001) with high among-study statistical heterogeneity (*I*^2^ = 76.2%; Tau^2^ = 0.3524) in the REM (Figure 4A).

##### 3.3.3.2. Pancreatic fistula

Pancreatic fistula was reported in 13 studies (1,938 RPD vs. 2,104 OPD) and was significantly lower in RPD (OR: 0.67; 95% CI: 0.55 to 0.82; *p* < 0.001) with low among-study statistical heterogeneity (*I*^2^ = 26.9%) in the FEM (Figure 4B).

##### 3.3.3.3. Delayed gastric emptying

Thirteen studies reported the results of delayed gastric emptying (1,055 RPD vs. 1,257 OPD). No statistically significant differences were found between the two groups (OR: 0.67; 95% CI: 0.38 to 1.18; *p* = 0.165), with a high heterogeneity (*I*^2^ = 51.3%, Tau^2^ = 0.4918) in the REM (Figure 4C).

##### 3.3.3.4. Length of stay

20 studies reported the data of length of stay (2,496 RPD vs. 3,220 OPD). The meta-analysis showed OPD has significant longer length of stay than RPD (WMD: −1.90; 95% CI: −2.47 to −1.33; *p* < 0.001), with a high heterogeneity (*I*^2^ = 68.5%, Tau^2^ = 0.6432) in the REM (Figure 4D).

##### 3.3.3.5. 90-day Mortality

12 studies reported the data of 90-day mortality (1,841 RPD vs. 2,591 OPD). The meta-analysis showed RPD has significant lower 90-day mortality than OPD (OR: 0.65; 95% CI: 0.45 to 0.95; *p* = 0.025), with a low heterogeneity (*I*^2^ = 3.8%) in the FEM (Figure 4E).

## 4. Discussion

Since first RAPD was reported in 2007, RPD technology has developed rapidly. With the improvement of equipment and doctors gradually through the learning curve, the safety and efficiency of RPD comparing to OPD is gradually improved. Hence, the relevant research results have timeliness. Therefore, although there have been previous meta-analyses comparing clinical outcomes between OPD and RPD, these meta-analyses contained some former studies and can't sufficiently represent current situation. So, we screened articles published after 2016 in our meta-analyses and contained several new studies in this year in order to show the latest RPD development as far as possible.

### 4.1. Findings in our meta-analyses

According to the result of our meta-analysis, RPD has a longer operative time and lower blood loss comparing to OPD, which is also supported by previous researches. As a significant advantage of robotic surgery, RPD showed a lower blood loss. And it may be explained by high-quality three-dimensional (3-D), optical 10–15 magnification vision, and greater precision (25). Multiple factors may lead to the longer operative time in RPD. On one hand, the long time for preparation of machine before operation resulted in a longer operative time. On the other hand, surgeons in these studies not passing through the learning curve may also contribute to longer operative time. What deserve attention is that the result of operative time and estimated blood loss showed high heterogeneity. According to the forest plot of operative time (Figure 2A), most studies raised up that operative time was higher in RPD group, but four studies reached the opposite conclusion (7, 14, 15, 19). Many factors can affect the operation time, of which the most important factor is the proficiency of the surgeon. In addition, the equipment of the center and the surgery team also influence the operative time. The heterogeneity of estimated blood loss was also high (*I*^2^ = 0.927). One study showed obvious different conclusion comparing with other studies (7). It's obvious that the exclusion of this study will not influence the conclusion of our article. Blood loss can be affected by the proficiency of the surgeon and the condition of the patients (e.g., the location and kind of cancer).

Lymph nodes harvested and margin status are considered to be consistent with prognosis of pancreatic cancer. Although various methods of margin quantification in different studies increase the complexity to assessment, margin status is still recognized to have prognostic significance for overall survival of pancreatic ductal adenocarcinoma (PDAC) in PD (26). Similarly, the number of lymph nodes harvested also plays a role in the reveal of prognostic performance (27). Historically, a mass of researches on OPD and RPD compared their differences in margin status, and previous meta-analysis also counted the oncological outcomes of the two groups. Except the meta-analysis of Dong et al. demonstrated that the RPD group has a larger number of lymph nodes harvested and a lower resection margin involvement rate, another two meta-analyses early and this meta-analysis all reveal that there is no difference of those oncological outcomes in the two groups (28–30). The heterogeneity of lymph nodes harvested and overall complication rate is high. The composition of patients’ tumor varies in different studies, which may lead to the heterogeneity of lymph nodes harvested. Besides, different operation centers may have different diagnostic criteria and definition for post-operative complications, causing the heterogeneity of overall complication rate.

Furthermore, we analyzed the oncological outcomes in studies limited to pancreatic cancer. Five studies analyzed patients with only pancreatic cancer, and other studies contained patients with kinds of disease which accepted RPD or OPD. In most studies, patients accepted PD because of different diseases, including pancreatic cancer, ampullary adenocarcinoma, and neuroendocrine tumor. Obviously, the malignancy of these tumors is different, reducing the credibility of the comparison of the prognosis indicator between OPD and RPD.

Of the five studies limited to pancreatic cancer, four involved lymph nodes harvested, and analysis of these four articles also showed no difference in RPD and OPD groups. Only two articles limited in pancreatic cancer mentioned R0 resection which is too few to analyze. Comparing the results of the two meta-analyses, no different conclusions were reached.

The safety of RPD has been proved in previous studies. As expected, our meta-analysis revealed that clinical outcomes favor RPD, including overall complication rates, pancreatic fistula rate, and length of hospital stay. Besides, different from the previous meta-analysis, this meta-analysis demonstrated that 90-day mortality also favors RPD.

### 4.2. Strengths

The safety and efficiency of RPD comparing to OPD is gradually improved, owing to the improvement of equipment and doctors gradually through the learning curve. This is the latest meta-analyses that included all eligible studies published in these 5 years. The number of studies is one strength of our article. Furthermore, to our knowledge, this is the first meta-analyses and systematic review that included all studies limited to patients with pancreatic cancer.

### 4.3. Limitations

Although we found 5 studies researching RPD and OPD in pancreatic cancer patients, most studies mix patients with various diseases together for analysis, making it impossible to conduct subgroup analysis. Besides, lack of RCTs in our meta-analysis is another limitation.

### 4.4. Implications for clinical practice

This meta-analysis found that RPD showed lower blood loss, overall complication rates, pancreatic fistula, and 90-day mortality compared with OPD. Besides, length of hospital stay was shorter in RPD. Although the operative time is longer in RPD group, and there were no differences in R0 resection and lymph nodes harvested, RPD has shown benefits over OPD and seemed to be proposed as an equivalent alternative to OPD. However, all the current studies about OPD and RPD are not RCTs, and high-quality studies are still needed. In addition, centers with the ability to perform a sufficient number of surgeries and professional surgeons who have overcome the learning curve are essential for successful implementation of RPD. What's more, RPD costs much higher than OPD, which is also an important factor in the choice of surgical methods.

## Data Availability

The original contributions presented in the study are included in the article/**Supplementary Material**, further inquiries can be directed to the corresponding author/s.
