# Supplementary material for: Meta-analysis of robotic versus open pancreaticoduodenectomy in all patients and pancreatic cancer patients
Source: Front Surg. 2022 Oct 11;9:989065. doi: 10.3389/fsurg.2022.989065 (PMC9592922; doi:10.3389/fsurg.2022.989065)
Supplement: Supplementary file 1 [file Table1.docx]

| Article | Selection | | | | Comparability | Outcome | | | Total |
| --- | --- | --- | --- | --- | --- | --- | --- | --- | --- |
|  | 1 | 2 | 3 | 4 | 1 | 1 | 2 | 3 |  |
| Emanuele 2018 | 1 | 1 | 1 | 0 | 0 | 1 | 1 | 1 | 6 |
| Hassan. 2021 | 1 | 1 | 1 | 0 | 2 | 1 | 1 | 1 | 8 |
| Shyr 2021 | 1 | 1 | 1 | 0 | 2 | 1 | 0 | 0 | 6 |
| Maria 2020 | 1 | 1 | 1 | 0 | 2 | 1 | 1 | 0 | 7 |
| Weng 2020 | 1 | 1 | 1 | 0 | 2 | 1 | 1 | 1 | 8 |
| Amer 2016 | 1 | 1 | 1 | 0 | 0 | 1 | 1 | 1 | 6 |
| Matthew 2016 | 1 | 0 | 1 | 0 | 2 | 1 | 1 | 1 | 7 |
| Mejia 2020 | 1 | 1 | 1 | 0 | 0 | 1 | 1 | 1 | 6 |
| Wang 2018 | 1 | 1 | 1 | 0 | 2 | 1 | 1 | 1 | 8 |
| Kim 2018 | 1 | 1 | 1 | 0 | 2 | 1 | 1 | 0 | 7 |
| Varley 2018 | 1 | 1 | 1 | 0 | 1 | 1 | 1 | 1 | 7 |
| Cai 2019 | 1 | 1 | 1 | 0 | 2 | 1 | 1 | 1 | 8 |
| Paolini 2021 | 1 | 1 | 1 | 0 | 0 | 1 | 1 | 1 | 6 |
| Benedetto 2018 | 1 | 1 | 1 | 0 | 2 | 1 | 0 | 1 | 7 |
| Marino 2019 | 1 | 1 | 1 | 0 | 2 | 1 | 1 | 1 | 8 |
| Shi 2021 | 1 | 1 | 1 | 0 | 2 | 1 | 1 | 1 | 8 |
| Bencini 2020 | 1 | 1 | 1 | 0 | 2 | 1 | 1 | 1 | 8 |
| Hyeyeon 2020 | 1 | 1 | 1 | 0 | 2 | 1 | 0 | 1 | 7 |
| Oosten 2020 | 1 | 1 | 1 | 0 | 2 | 1 | 1 | 1 | 8 |
| Shyr 2020 | 1 | 1 | 1 | 0 | 0 | 1 | 1 | 1 | 6 |
| Wang 2021 | 1 | 1 | 1 | 0 | 1 | 1 | 1 | 1 | 7 |

NOS scale for case control studies

**Selection**

1) Is the case definition adequate?

a) yes, with independent validation Ø

b) yes, eg record linkage or based on self reports

c) no description

2) Representativeness of the cases

a) consecutive or obviously representative series of cases Ø

b) potential for selection biases or not stated

3) Selection of Controls

a) community controls Ø

b) hospital controls

c) no description

4) Definition of Controls

a) no history of disease (endpoint) Ø

b) no description of source

**Comparability**

1) Comparability of cases and controls on the basis of the design or analysis

a) study controls for _______________ (Select the most important factor.)

b) study controls for any additional factor Ø (This criteria could be modified to indicate specific control for a second important factor.)

**Exposure**

1) Ascertainment of exposure

a) secure record (eg surgical records) Ø

b) structured interview where blind to case/control status Ø

c) interview not blinded to case/control status

d) written self report or medical record only

e) no description

2) Same method of ascertainment for cases and controls

a) yes Ø

b) no

3) Non-Response rate

a) same rate for both groups Ø

b) non respondents described

c) rate different and no designation
