# Supplementary material for: Meta-analysis of robotic versus open pancreaticoduodenectomy in all patients and pancreatic cancer patients
Source: Front Surg. 2022 Oct 11;9:989065. doi: 10.3389/fsurg.2022.989065 (PMC9592922; doi:10.3389/fsurg.2022.989065)
Supplement: Supplementary file 2 [file Table2.docx]

| Article | Country | Design | Number  of patients  (Robotic  ) | Number  of patients  (Open  ) | Quality score | Robotic technique | Indication for  surgery (benign  or malignant  disease) |
| --- | --- | --- | --- | --- | --- | --- | --- |
| Emanuele 2018 | Italy | Retrospective | 24 | 26 | 6 | RPD | M |
| Hassan. 2021 | America | Retrospective | 310 | 310 | 8 | RPD | M |
| Shyr 2021 | China | Prospective | 65 | 65 | 6 | RPD | M |
| Maria 2020 | America | Retrospective | 38 | 38 | 7 | RPD | M |
| Weng 2020 | China | Retrospective | 105 | 210 | 8 | RAPD | M |
| Amer 2016 | America | Retrospective | 211 | 817 | 6 | RPD | B&M |
| Matthew 2016 | America | Retrospective | 152 | 152 | 7 | RPD | B&M |
| Mejia 2020 | America | Retrospective | 102 | 54 | 6 | RPD | B&M |
| Wang 2018 | China | Prospective | 87 | 87 | 8 | RPD | B&M |
| Kim 2018 | Korea | Retrospective | 51 | 186 | 7 | RPD | B&M |
| Varley 2018 | America | Retrospective | 133 | 149 | 7 | RPD | B&M |
| Cai 2019 | America | Prospective | 460 | 405 | 8 | RPD | B&M |
| Paolini 2021 | Italy | Retrospective | 65 | 53 | 6 | RPD | B&M |
| Benedetto 2018 | Spain | Prospective | 17 | 17 | 7 | RPD | B&M |
| Marino 2019 | Italy | Prospective | 35 | 35 | 8 | RAPD | B&M |
| Shi 2021 | China | Retrospective | 187 | 187 | 8 | RAPD | B&M |
| Bencini 2020 | Italy | Retrospective | 35 | 35 | 8 | RPD | B&M |
| Hyeyeon 2020 | Korea | Retrospective | 55 | 55 | 7 | RAPD | B&M |
| Oosten 2020 | America | Retrospective | 96 | 192 | 8 | RPD | B&M |
| Shyr 2020 | China | Retrospective | 284 | 169 | 6 | RPD | B&M |
| Wang 2021 | China | Prospective | 49 | 43 | 7 | RPD | B&M |
