# Supplementary material for: Meta-analysis of robotic versus open pancreaticoduodenectomy in all patients and pancreatic cancer patients
Source: Front Surg. 2022 Oct 11;9:989065. doi: 10.3389/fsurg.2022.989065 (PMC9592922; doi:10.3389/fsurg.2022.989065)
Supplement: Supplementary file 3 [file Table3.docx]

| Article | Age (years) | | Gender (male) (%) | | BMI | | Tumor diameter (cm) | | Preoperative CA 199 | |
| --- | --- | --- | --- | --- | --- | --- | --- | --- | --- | --- |
|  | RPD | OPD | RPD | OPD | RPD | OPD | RPD | OPD | RPD | OPD |
| Emanuele 2018 | 65 (58.5–74.75) | 72.5 (59.75–78.75) | 50.0 | 54.1 | 23.1± 3.2 | 24.1±3.1 | 2.7± 0.6 | 2.7±0.9 | 353.3± 528.6 | 1362.7±4497 |
| Hassan. 2021 | 66±21.3 | 68.1± 19.3 | 50.1 | 51.1 | NM | NM | NM | NM | NM | NM |
| Shyr 2021 | 66±13 | 66±11 | 52.3 | 40.0 | 24±4 | 22±3 | 3.1±0.8 | 3.1±0.7 | NM | NM |
| Maria 2020 | 66 (38–84) ^*^ | 68 (42–81) ^*^ | 42.1 | 42.1 | 24.7 (19.6–39.1) ^*^ | 25.7 (15.8–44.8) ^*^ | 3 (0.5–6) ^*^ | 2.9 (0.9–7) ^*^ | NM | NM |
| Weng 2020 | 63 (57–68) | 64 (58–70) | 61.7 | 65.9 | 22.8±2.8 | 22.6±3.1 | 3 (2.2–3.5) | 3.0 (2.3–3.8) | 144.4 (40.1–375.4) | 153.4 (46.0–505.2) |
| Amer 2016 | 67 (15–86) ^*^ | 65 (15–93) ^*^ | 52.9 | 55.5 | 27.5 (18.1–47.6) ^*^ | 26.1 (14.7–85.5) ^*^ | 2.5 (0.1–26.0) ^*^ | 2.9 (0–5.0) ^*^ | NM | NM |
| Matthew 2016 | NM | NM | NM | NM | NM | NM | NM | NM | NM | NM |
| Mejia 2020 | 66±10.6 | 61.7±14.1 | 52 | 55.6 | NM | NM | 3.4±1.6 | 3.7±2.1 | NM | NM |
| Wang 2018 | NM | NM | 50 | 56.7 | NM | NM | NM | NM | NM | NM |
| Kim 2018 | 60.7 ± 11.9 | 65.4 ± 10.1 | 47.1 | 58.1 | 22.7 ± 2.5 | 24.0 ± 3.1 | NM | NM | NM | NM |
| Varley 2018 | 66.3 ± 10.6 | 67.0 ± 10.5 | 48 | 53 | 27.5 ± 6.1 | 26.7 ± 5.6 | NM | NM | NM | NM |
| Cai 2019 | 66.5 ± 11.0 | 67.5 ± 10.7 | 55 | 52.1 | 27.8 ± 5.8 | 27.2 ± 5.9 | NM | NM | NM | NM |
| Paolini 2021 | 70 (42–85) ^*^ | 73 (45–91) ^*^ | 50.9 | 53.8 | 26 (17–33) ^*^ | 23 (14–33) ^*^ | 2.3 (0.7–6) ^*^ | 2.5 (0.6–8.2) ^*^ | 85.0 (1.6–1617.0) ^*^ | 132.3 (1.6–91000.0) ^*^ |
| Benedetto 2018 | 66.8±9.5 | 61.4±11.9 | 47.1 | 58.8 | 23.8± 4.1 | 24.6±3.36 | 24.1±5.4 | 24.8±6.1 | NM | NM |
| Marino 2019 | 60.4 (43–72) ^*^ | 62.3 (45–73) ^*^ | 54.3 | 42.9 | 23.8 (19.4–30.9) ^*^ | 23.5 (18.8–28.1) ^*^ | 2.35 (1.6–3.4) ^*^ | 2.22 (1.2–3.5) ^*^ | NM | NM |
| Shi 2021 | 60.9±11.4 | 60.1±10.8 | 58.3 | 57.2 | NM | NM | 2.7±1.1 | 2.7±1.3 | NM | NM |
| Bencini 2020 | 70.5 (42–85) ^*^ | 69 (50–88) ^*^ | 56.3 | 45.7 | 26 (18–32) ^*^ | 24 (18–38) ^*^ | 30 (18–40) ^*^ | 37 (2–51) ^*^ | 143 (2–1617) ^*^ | 70 (2–2617) ^*^ |
| Hyeyeon 2020 | 58.6±8.3 | 59.9±13.4 | 47.3 | 54.5 | 23.7±2.8 | 23.9±3.6 | 2.6±1.2 | 2.6±1.8 | NM | NM |
| Oosten 2020 | 67 (60-73) | 67 (58-73) | NM | NM | 26 (23-30) | 27 (23-29) | NM | NM | NM | NM |
| Shyr 2020 | 65± 12 | 64± 11 | 53.3 | 53.5 | 24± 4 | 23±3 | 3.2± 1.5 | 3.7±2.5 | NM | NM |
| Wang 2021 | 64.7 ± 11.8 | 64.8 ± 11.6 | 51.9 | 53.4 | 27.7 ± 5.6 | 27.4 ± 5.8 | NM | NM | NM | NM |

Table 2. Comparison of patients’ baseline characteristics in robotic vs. open pancreaticoduodenectomy. BMI: body mass index;

Expressed in mean ± SD & median(IQR)

*: Expressed in median (range)
