# Supplementary material for: Meta-analysis of robotic versus open pancreaticoduodenectomy in all patients and pancreatic cancer patients
Source: Front Surg. 2022 Oct 11;9:989065. doi: 10.3389/fsurg.2022.989065 (PMC9592922; doi:10.3389/fsurg.2022.989065)
Supplement: Supplementary file 4 [file Table4.docx]

| Outcomes | Studies, n | RPD | OPD | WMD/OR (95% CI) | P value | Heterogeneity | | |
| --- | --- | --- | --- | --- | --- | --- | --- | --- |
|  |  |  |  |  |  | I^2^ | | Tau^2^ |
| Intraoperative outcomes | | | |  |  |  |  | |
| Operative time | 17 | 1924 | 2690 | 64.60 (26.89 to 102.21) | 0.001 | 0.978 |  | |
| Estimated blood loss | 14 | 1604 | 1583 | -185.44 (-239.66 to -131.21) | <0.001 | 0.927 |  | |
| Oncological outcomes | | | |  |  |  |  | |
| Lymph nodes harvested | 13 | 1337 | 1699 | 1.13 (-0.27 to 2.54) | 0.115 | 0.828 | 4.69 | |
| R0 resection | 10 | 955 | 1026 | 1.02 (0.79 to 1.30) | 0.889 | 0 | n | |
| Post-operative outcomes | | | |  |  |  |  | |
| Overall complication rates | 13 | 1192 | 1856 | 0.66 (0.44 to 0.97) | <0.001 | 0.762 | 0.3524 | |
| Pancreatic fistula | 13 | 1938 | 2104 | 0.67 (0.55 to 0.82) | <0.001 | 0.269 | n | |
| Length of stay | 20 | 2496 | 3220 | -1.90 (-2.47 to -1.33) | <0.001 | 0.685 | 0.6432 | |
| 90-day mortality | 12 | 1841 | 2591 | 0.77 (0.45 to 0.95) | 0.025 | 0.038 | n | |
